# NASH and Systemic Complications: From Basic to Clinical Research

**DOI:** 10.3390/biomedicines9121913

**Published:** 2021-12-14

**Authors:** Sabine Baumgartner, Ronit Shiri-Sverdlov

**Affiliations:** 1 Department of Nutrition and Movement Sciences, School of Nutrition and Translational Research in Metabolism (NUTRIM), Maastricht University, Universiteitssingel 50, 6229 ER Maastricht, The Netherlands; 2 Department of Molecular Genetics, School of Nutrition and Translational Research in Metabolism (NUTRIM), Maastricht University, Universiteitssingel 50, 6229 ER Maastricht, The Netherlands

Nonalcoholic fatty liver disease (NAFLD) is known as the hepatic manifestation of the metabolic syndrome, and while most patients develop simple steatosis, up to one-third can develop nonalcoholic steatohepatitis (NASH). NASH is a chronic inflammatory condition of the liver that can further progress to fibrosis and cirrhosis, which may eventually lead to liver failure and death. In this Special Issue, 12 scientific contributions, including nine reviews and three original research articles, address basic and clinical research on NASH and its systemic complications. Articles were divided into three subtopics: (1) mechanistic tools to investigate all disease aspects of NAFLD; (2) extrahepatic manifestations of NASH; and (3) promising biomarkers to diagnose NASH and interventions to treat NASH.

Currently, the mechanisms by which inflammation is triggered within the context of NASH are unknown. Consequently, therapeutic options are poor, non-invasive markers to detect NASH do not exist and the long-term burden to society is constantly increasing. Ultimately, there is an urgent need for a physiological “humanized” model that displays a liver phenotype identical to human disease, including all stages and systemic characteristics. In this Special Issue, Oligschläger et al. [1] provide an overview on the currently existing murine models and describe the advantages, limitations and challenges in using each model. While murine models are considered indispensable tools for studying chronic liver disease pathology, the majority of the existing models do not demonstrate all the disease stages or do not have the systemic characteristic of NASH. Therefore, selection of the model is highly dependent on the research question. Furthermore, as NASH progression greatly varies across different strains, it is often not sufficient to include only one model in each study. Considering the ethical issues related to the use of animals in research, the high costs and the low success rate of clinical trials which are based on murine models, great effort has been put to develop surrogate to the animal testing. One of the most promising recent and cost-effective alternative approaches is to employ machine learning and artificial intelligence on organ-on-a-chip technology. Such a high-throughput approach allows for the early identification of mechanisms leading to NASH and the assessment of the efficacy and toxicity of novel treatments. De Chiara et al. [2] provide a wide overview of the new implementation of organ-on-a-chip into the field of NAFLD, the current findings and limitations. They further elaborate on the multiple opportunities which will enable us to drastically limit the use of preclinical models and accelerate translational research in the field of NASH. Recently, organ-on-a-chip has also been used as a model to investigate the complex mechanisms which leads to the transition to hepatocellular carcinoma (HCC). In this Special Issue, Ramai et al. [3] review the pathogenesis of NASH leading to HCC and emerging pathological concepts. These mechanisms include the effects of cellular, genetic, immunologic, metabolic, and endocrine pathways. Combing artificial intelligence with complex organ-on-a-chip tools will allow us to investigate the interaction between these pathways in a rapid and accurate manner and aid the development of personalized treatment to NASH, as well as HCC patients, as organ-on-a-chip technologies need to be further developed before they will lead to the identifications of early and reliable diagnostic methods. In the meanwhile, extensive efforts are put to establishing imaging modalities for the identification of individuals at risk to develop advanced stage of NASH. Currently there are several imaging modalities for the assessment of hepatic steatosis and fibrosis. The extensive use in these imaging modalities within the context of NASH is described in the review of Grat et al. [4]. However, identification of inflammation by these tools and predication of the chance for complications based on early images remains a challenge. The 13C-aminopyrine breath test (ABT) evaluates the microsomal liver function and could be a potential candidate to allow the discrimination between simple steatosis to NASH. Using this approach, in this Special Issue, Verlinden et al. [5] aimed to evaluate a potential change in liver function in NASH patients and to assess the diagnostic power to detect NASH. They demonstrated that the microsomal liver function of patients with NASH is significantly decreased, even in the absence of fibrosis. Nevertheless, while the 13C-aminopyrine breath test show promise in diagnosis, they currently do not allow for predication of local and extrahepatic systemic complications of NASH. Within this context, extracellular vesicles (EVs) have gained increased attention due to their function as mediators of cellular communication. In their review, Højland Ipsen et al. [6] provide an update on current experimental and clinical findings of the role of EVs in NASH progression and point towards the potential of EVs as disease markers and treatment targets. Within the same context, leptin, an adipokine involved in energy homeostasis and lipid metabolism, has also been investigated as a potential biomarker and as a target of treatment in the NAFLD spectrum. Jiménez-Cortegana et al. [7] describe that the use of leptin is debatable, since it has been shown to be an independent predictor of the presence or development of NAFLD, while it has also been associated with hepatic steatosis and could promote the induction of NASH or liver fibrosis in NAFLD patients. As a therapeutic option, the use of leptin is controversial due to its proinflammatory properties but does highlight that the development of leptin analogues and leptin sensitizers is promising. Overall, these recent developments in preclinical and surrogate models as well as non-invasive tools to assess NASH are expected to drastically accelerate the speed by which new knowledge regarding NASH progression is gained as well as the identification of novel pharmaceutical target(s) and accurate timing for treatment.

NAFLD comprises a wide range of liver disorders, from simple steatosis to NASH and—if not treated—life-threatening complications such as cirrhosis and HCC. In addition to effects on hepatic-related events, evidence also points towards a link of NAFDL/NASH with extra-hepatic complications, including central nervous system (CNS) diseases. In this Special Issue, Colognesi et al. [8] summarize the main correlations observed between NAFLD/NASH and CNS dysfunctions, such as depression, cognitive impairments, dementia and Alzheimer’s disease (AD), where an inflammatory state and oxidative stress both play a pivotal role. Even though there are conflicting results whether there is a causal relation between liver damage and the development of cognitive dysfunction, many signaling pathways are altered in both NAFLD/NASH and CNS dysfunction, suggesting that these diseases at least share a pathogenetic component. The pathology of NAFLD has also been linked to a lower microbial diversity and an altered gut barrier integrity, exposing the host to bacterial components and inducing immune responses and inflammation. Indeed, NASH patients have an increased intestinal permeability and a decreased bacterial overgrowth, which correlated with the severity of steatosis. In addition, several lines of evidence support increased intestinal permeability and bacterial translocation as possible causes in the development of metabolic disease, such as the metabolic syndrome and type 2 diabetes. As presented by Plaza-Díaz et al. [9] in this Special Issue, the potential role of alterations in gut microbiota in NASH as biomarker for prognosis and diagnosis is promising. Moreover, the involvement of microbiota as a potential target to treat for NAFLD and NASH by supplementation of prebiotics and probiotics and fecal microbiota transplantation is discussed.

Dietary imbalances are increasingly recognized as the root cause of NASH-related comorbidities and Simón et al. [10] describe in this Special Issue that a deficit in magnesium, a micronutrient found in most green vegetables, legumes, peas, beans and nuts, might aggravate NASH and its related diseases. The authors characterized the potential role that magnesium might play in the development of liver diseases and metabolic syndrome. They have demonstrated that magnesium supplementation is linked to reduced NASH-related mortality and future studies to investigate the potential of magnesium as a therapeutic approach are warranted. Therapeutic options to treat NAFLD and NASH are often targeted to improve glucose metabolism since dysregulations in glucose homeostasis are an important driver in the pathogenesis of NASH. Previous studies demonstrated beneficial effects of antiglycemic treatments in diabetic NASH patients, while not much is known concerning their effect in a non-diabetic setting. In this Special Issue, Hupa-Breier et al. [11] investigate the effect of long-acting GLP-1 agonist dulaglutide and sodium-glucose cotransporter 2 (SGLT-2) inhibitor empagliflozin and their combination in a non-diabetic mouse model of NASH. Even though this GLP-1 agonist and SGLT-2 inhibitor did not prevent hepatic steatosis, dulaglutide and the combination of dulaglutide with empagliflozin improved glucose homeostasis and induced anti-inflammatory and anti-fibrotic pathways, which could have important implications for the treatment of NASH. As described above, pharmacological therapy often addresses NASH-associated co-morbidities, while treatments targeting the liver itself are scarce. Mesenchymal stromal cell (MSC) transplantation has been shown to ameliorate hepatic lipid load, tissue inflammation and fibrosis in rodent animal models of NASH. However, the mechanisms of these MSCs are not known, which is crucial to improve their therapeutic potential. In this Special Issue, Hsu et al. [12] transplanted bone marrow-derived MSCs into the livers of a NASH mouse model. The diet that was used to induce NASH in this model was characterized by an impairment of the central carbon and amino acid metabolism, which was probably the trigger for mitochondrial and peroxisomal dysfunction. Transplanting MSCs in the host livers ameliorated lipid storage and associated perturbance of tissue homeostasis likely by donating healthy mitochondria to the hepatocytes, which provided oxidative capacity for lipid breakdown and thus recovery of liver tissue.

While we have increased our mechanistic knowledge regarding the pathogenesis of NASH within the last decade, treatment options are still limited and liver biopsies have remained the gold standard for diagnosis. To achieve major clinical breakthrough for NASH patients, it is not sufficient to use a single animal model since each model has specific limitations. Furthermore, we should rely more on alternative models such as organ-on-a-chip, which will enable us to explore unknown aspects of disease pathogenesis much faster and serve as clinically relevant surrogates for murine models. Another important direction to improve patient’s health is to pay more attention to extrahepatic, organ specific and systemic effects, which are associated with NASH. This Special Issue describes several metabolic disturbances related to NASH which should be monitored more closely in in preclinical studies and in patient populations. As demonstrated in this issue, the technological developments in disease models along with advanced mathematical data analysis and the view of NASH as a systemic disease, already generated new opportunities for the discovery of novel drugs and diagnostic tools to monitor diseases progression.

In summary, the articles in this Special Issue include an up-to-date overview of the rapidly developing technologies, novel targets for intervention and insights in the field in NASH. Additionally, these articles describe the major challenges in the field, strategies to overcome them and suggestions for future directions. To improve patient’s outcome, clinicians, as well as scientists with biomedical, nutrition, physics and mathematics backgrounds, should join forces. Although challenges remain, the future of the field seems promising as these novel technologies and developments are expected to lead to progress in NASH.
